# Activation of the peripheral immune system regulates neuronal aromatase in the adult zebra finch brain

**DOI:** 10.1038/s41598-017-10573-x

**Published:** 2017-08-31

**Authors:** Alyssa L. Pedersen, Cassie J. Gould, Colin J. Saldanha

**Affiliations:** American University, Department of Biology & Behavior, Cognition & Neuroscience program, Washington, DC 20016 USA

## Abstract

Estradiol provision via neural aromatization decreases neuro-inflammation and –degeneration, but almost nothing is known about the interactions between the peripheral immune system and brain aromatase. Given the vulnerability of the CNS we reasoned that brain aromatization may protect circuits from the threats of peripheral infection; perhaps shielding cells that are less resilient from the degeneration associated with peripheral infection or trauma. Lipopolysaccharide (LPS) or vehicle was administered peripherally to adult zebra finches and sickness behavior was recorded 2 or 24 hours later. The central transcription of cytokines and aromatase was measured, as were telencephalic aromatase activity and immunoreactive aromatase (24 hour time point only). Two hours post LPS, sickness-like behaviors increased, the transcription of IL-1β was higher in both sexes, and TNFα was elevated in females. 24 hours post-LPS, the behavior of LPS birds was similar to controls, and cytokines had returned to baseline, but aromatase mRNA and activity were elevated in both sexes. Immunocytochemistry revealed greater numbers of aromatase-expressing neurons in LPS birds. These data suggest that the activation of the immune system via peripheral endotoxin increases neuronal aromatase; a mechanism that may rapidly generate a potent anti-neuroinflammatory steroid in response to peripheral activation of the immune system.

## Introduction

Estrogens are established modulators of vertebrate anatomy and physiology throughout the lifespan. Estradiol (E_2_) has dramatic organizational effects on the developing CNS in birds and mammals^1–3^, but also activates neural pathways in juveniles and adults. Earlier work demonstrated a profound role for E_2_ in masculinization of the brain and activation of copulatory and aggressive behaviors^4–6^. More recently, the range of physiological processes affected by E_2_ has broadened considerably and now includes learning, memory, pain, balance, and mood, among others^7–14^.

Part of this expansion includes a link between E_2_ and inflammatory signaling. Post-menopausal women who are not on hormone replacement therapy have higher indices of inflammation compared to premenopausal women^11^. This is likely due to a loss of circulating E_2_ since the administration of this estrogen decreases circulating cytokine levels in mice, and microglial activation following lipopolysaccharide (LPS) *in vitro* ^15–17^. Finally, tolerance to LPS is pronounced in rodents when challenged during proestrus (when E_2_ is high) relative to diestrus^18^. Thus, circulating E_2_ appears to be a potent neuroprotective signal involving (anti-) inflammatory pathways in multiple vertebrate tissues in the periphery.

In addition to the influence of plasma E_2_ mentioned above, work in our laboratory shows E_2_ may also be a potent neuroprotective signal involving (anti-) inflammatory pathways in the brain. Specifically, inhibition of aromatase and concomitant replacement of E_2_ respectively increases and decreases the central expression of cytokines TNFα and IL-1β, and cox-2/Prostaglandin E2 (PGE2) following brain injury^19^. Brain-derived E_2_ is anti-inflammatory in other models as well, including mouse models of global cerebral ischemia^20^ and LPS^21^, and seems to be mediated by ERα^21^.

E_2_ appears to be neuroprotective following brain injury. Local aromatase inhibition increases and E_2_ administration decreases, the extent of damage^22, 23^, apoptosis^22^, cyto- and neurogenesis^24^. Zebra finches, in particular, seem to have a dramatic response to E_2_, perhaps due to the abundant localization of aromatase throughout the telencephalon and diencephalon^25^. Zebra finches do not show the wave of secondary degeneration following brain injury that is prominent in mammalian models^26^. Interestingly, this wave of secondary degeneration is only evident following aromatase inhibition. Thus, finches appear to possess a powerful mechanism that allows the brain to be protected following damage, and E_2_ may play a large role in regulating cell turnover and inflammatory signaling following insult.

Interestingly, there is good reason to hypothesize the reciprocal relationship as evidence suggests that inflammatory pathways may regulate neural aromatase expression. Cytokine administration increases aromatase expression in breast cancer cells *in vitro* suggesting an interaction between inflammatory and steroidogenic pathways in peripheral tissues^27–29^. In neonatal rats, peripheral or central PGE2 administration increases cerebellar aromatase activity and E_2_ content *in vivo*, an effect that has permanent effects on the dendritic morphology and neurophysiology of Purkinje neurons^30^. Additionally, application of phytohaemagglutinin (PHA), directly to the brain of adult zebra finches is sufficient to induce a neuro-inflammatory response detectable via increases in central cytokines IL-1β and IL-6 and aromatase^31^. Notably, the increase of cytokine signaling occurs prior to the induction of injury-induced aromatase, suggesting that presence of inflammatory signaling following neural insult may serve as a biological signal to induce and/or regulate aromatase expression in the zebra finch. Indeed, we have recently shown that injury to the zebra finch brain in the presence of a cyclooxygenase inhibitor, mitigates the induction of aromatase and consequent E_2_ content, relative to injury in the presence of vehicle alone^32^. Thus, the inflammatory response which accompanies brain trauma appears to itself be key in the induction of central E_2_ synthesis. This elevation in E_2_ appears to serve as a potent anti-inflammatory agent that may protect the brain from further degeneration^19^.

Given the inductive role of inflammatory signals on central aromatization, and the subsequent anti-inflammatory effects of aromatization during brain trauma mentioned above, we considered the possibility of a broader interaction of inflammatory and steroidogenic pathways. We hypothesized that activation of the peripheral immune system might affect neural aromatase expression, thereby increasing central E_2_. The induction of neuronal aromatase and E_2_ synthesis may limit neuroinflammatory signaling, a potentially damaging consequence of systemic inflammation or infection. Therefore, E_2_ via aromatization may limit potentially severe costs of sickness and protect vulnerable tissues such as the brain from dramatic and perhaps irreversible damage. The brain is particularly vulnerable to insult following systemic infections^33–35^. A systemic infection produces cytokines that circulate through the blood and communicate with the brain, via the activation of resident microglial populations, and can occur without disruption of the blood brain barrier ^33–35^. This is particularly problematic for central neurons, a cell-type with lower rates of cell-turnover, particularly neurogenesis, in adults of the more recently evolved vertebrates^36–38^. Peripheral inflammatory signaling can intensify symptoms and promote progression of many neurodegenerative diseases or disorders such as Alzheimer’s disease, dementia, depression, and head injury^35^.

To test if peripheral activation of the immune system induces aromatase and regulates its distribution, we delivered a peripheral injection of lipopolysaccharide (LPS) to adult zebra finches of both sexes and measured the transcription, biochemical activity, neural distribution, and cell-specific expression of central aromatase.

## Results

### Body Weight

LPS birds lost significantly more body weight compared to controls. Analyses revealed a main effect of treatment (*F* (1,8) = 28.9, *p* < 0.01), with no other sources of significance (sex: (*F* (1,8) = 3.21, *p* = 0.1); sex x treatment (*F* (1,8) = 0.10, *p* = 0.75). The main effect of treatment is driven by LPS birds losing more weight compared to control birds, regardless of sex.

### Behavior

#### Pre - injection

There was no difference between the LPS animals and control animals when analyzing their behavior prior to injection. No significant main effects or interactions were detected for hops (treatment: *F* (1,16) = 0.02, *p* = 0.96; sex: *F* (1,16) = 0.005, *p* = 0.94; treatment x sex: *F* (1,16) = 0.17, *p* = 0.68), flights (treatment: *F* (1,16) = 2.68, *p* = 0.12; sex: *F* (1,16) = 0.46, *p* = 0.50; treatment x sex: *F* (1,16) = 0.01, *p* = 0.90), or resting behavior (treatment: *F* (1,16) = 0.1.50, *p* = 0.23; sex: *F* (1,16) = 0.81, *p* = 0.81; treatment x sex: *F* (1,16) = 1.45, *p* = 0.24).

#### 2-hours post-injection

LPS decreased locomotor behaviors compared to control 2-hours post-injection. For hops, there was a main effect of treatment (*F* (1,16) = 14.70, *p* < 0.001) but no other significant main effects or interaction (sex: *F* (1,16) = 0.46, *p* = 0.50; treatment x sex: *F* (1,16) = 0.01, *p* = 0.90). Post-hoc analyses revealed that the main effect was due to both males and females having higher hops in control compared to LPS treatment animals. Similarly, LPS decreased flight behavior. There was a main effect of treatment (*F* (1,16) = 16.70, *p* < 0.001), with no other significant main effect or interaction (sex: *F* (1,16) = 4.65, *p* = 0.06; treatment x sex: *F* (1,16) = 4.0, *p* = 0.06). Post-hoc analyses revealed that the main effect was due to both males and females having higher hops in control vs. LPS treatment animals. Finally, LPS treatment increased resting behavior. There was a main effect of treatment (*F* (1,16) = 629, *p* < 0.001), sex (*F* (1,16) = 29.1, *p* < 0.001), and a treatment by sex interaction *F* (1,16) = 30.0, *p* < 0.001). Post-hoc analyses revealed that LPS increased resting behavior for both males and females compared to controls. The main effect of sex and the sex by treatment interaction is driven by females spending more time resting compared to males, regardless of treatment.

#### 24-hours post-injection

Identical to the pre-observational data, there was no difference between the LPS and control animals 24-hours post injection. No significant main effects or interactions were found for hops (treatment: *F* (1,16) = 0.003, *p* = 0.95; sex: *F* (1,16) = 0.14, *p* = 0.71; treatment x sex: *F* (1,16) = 0.3.18, *p* = 0.09), flights (treatment: *F* (1,16) = 1.64, *p* = 0.21; sex: *F* (1,16) = 0.46, *p* = 0.50; treatment x sex: *F* (1,16) = 0.04, *p* = 0.83), or resting behavior (treatment: *F* (1,16) = 0.1.50, *p* = 0.23; sex: *F* (1,16) = 0.81, *p* = 0.81; treatment x sex: *F* (1,16) = 2.40, *p* = 0.33). Behavioral data from this experiment are shown in Fig. 2.

### Quantitative Polymerase-Chain Reaction

#### 2-hours post injection

*TNF-α*. TNF-α transcription varied significantly across treatment (*F* (1,24) = 9.20, *p* < 0.001), but no other significant main effect or interaction (sex: *F* (1,24) = 0.20, *p* = 0.65; treatment x sex: (*F* (1,24) = 2.12, *p* = 0.15). The main effect of treatment is driven by LPS females having higher levels of TNF-α mRNA compared to controls.

*IL-1β*. LPS increased IL-1β transcription, with a main effect of treatment (*F* (1,24) = 14.30, *p* = 0.005), but no other significant main effect or interaction (sex: *F* (1,24) = 3.66, *p* = 0.06; treatment x sex: (*F* (1,24) = 0.65, *p* = 0.42). The main effect of treatment is driven by both males and females having higher levels of IL-1β mRNA following LPS treatment compared to control treatment.

*IL-6*. In contrast to other cytokines, LPS did not increase IL-6 transcription (treatment: (*F* (1,24) = 1.21, *p* = 0.28). However, there was a main effect of sex (*F* (1,24) = 88.1, *p* < 0.001), with no treatment x sex interaction (*F* (1,24) = 0.38, *p* = 0.59). The main effect of sex is driven by females having higher levels of IL-6, regardless of treatment.

*Aromatase*. Aromatase transcription varied significantly across sex 2-hours post injection (*F* (1,24) = 16.3, *p* < 0.001), but with no main effect of treatment or an interaction between the two (treatment: *F* (1,24) = 1.15, *p* = 0.29; treatment x sex: (*F* (1,24) = 0.86, *p* = 0.36). The main effect of sex is driven by females having higher aromatase mRNA compared to males, regardless of treatment. Data from this experiment has been transformed into fold change and is depicted in Fig. 3. Means + SEM of ΔCt from this experiment are presented in Table 1.

**Table 1:** Levels of TNF-α, IL-1β, IL-6 and aromatase mRNA relative to Glyceraldehyde 3-phosphate dehydrogenase (GAPDH; ΔCt values + SEM) in adult male and female zebra finches (where lower number equals higher expression).

Gene target	Male control	Male LPS	Female control	Female LPS
**2-hours post-injection**				
TNF-α	7.48 ± 0.11	7.01 ± 0.22	**7.64 ± 0.09**	**6.89 ± 0.21**
IL-1β	**10.52 ± 0.33**	**9.10 ± 0.32**	**9.68 ± 0.23**	**8.76 ± 0.23**
IL-6	9.60 ± 0.11	9.50 ± 0.20	7.68 ± 0.27	7.31 ± 0.25
Aromatase	9.67 ± 0.53	9.61 ± 0.32	8.56 ± 0.28	7.85 ± 0.20
**24-hours post-injection**				
TNF-α	8.26 ± 0.16	7.66 ± 0.21	8.52 ± 0.25	8.75 ± 0.33
IL-1β	10.23 ± 0.32	10.03 ± 0.23	8.02 ± 0.44	8.0 ± 0.32
IL-6	9.74 ± 0.63	9.91 ± 0.65	9.56 ± 0.17	9.24 ± 0.23
Aromatase	**9.60 ± 0.29**	**8.58 ± 0.13**	**9.57 ± 0.12**	**8.23 ± 0.27**

Bolded values indicate significant difference between LPS and control animals within each sex.

#### 24-hours post injection

*TNF-α*. 24-hours post injection, there was a main effect of sex (*F* (1,24) = 7.58, *p* = 0.01), but no other significant main effect or interaction (treatment: *F* (1,24) = 0.53, *p* = 0.47; treatment x sex: (*F* (1,24) = 2.83, *p* = 0.10). The main effect of sex is driven by males having elevated levels of TNF-α mRNA 24-hours post treatment, but not females.

*IL-1β*. Similar to above, there was a main effect of sex (*F* (1,24) = 61.4, *p* < 0.001), but no other significant main effect or interaction (treatment: *F* (1,24) = 3.34, *p* = 0.08; treatment x sex: (*F* (1,24) = 3.17, *p* = 0.09). The main effect of sex is due to females having higher levels of IL-1β mRNA compared to males, regardless of treatment.

*IL-6*. No significant effects on IL-6 were found 24-hours post injection (treatment (*F* (1,24) = 0.02, *p* = 0.87); sex: (*F* (1,24) = 0.80, *p* = 0.37); treatment x sex: (*F* (1,24) = 0.26, *p* = 0.61).

*Aromatase*. In contrast to cytokine transcription, LPS increased aromatase transcription at 24 hours, with a main effect of treatment (*F* (1,24) = 30.2, *p* < 0.001), but with no main effect of sex or an interaction between the two (sex: *F* (1,24) = 0.74, *p* = 0.38; treatment x sex: (*F* (1,24) = 0.56, *p* = 0.46). The main effect of treatment is driven by both males and females having increased aromatase mRNA following LPS compared to controls. Data from this experiment has been transformed into fold change and is depicted in Fig. 3. Means + SEM of ΔCt from this experiment are presented in Table 1.

#### Pearson correlation

To examine if there is a positive relationship between 2-hour cytokine mRNA and 24-hour aromatase mRNA, we performed a set of correlations. Control TNF- *α* or IL-1β was not correlated with aromatase mRNA (TNF- *α*: *r*(14) = 0.21, *p* = 0.45; IL-1β: *r*(14) = 0.20, *p* = 0.47). LPS treated animals cytokine data was correlated with aromatase mRNA, but only for TNF- *α*. High TNF- *α* mRNA was positively correlated with aromatase mRNA at 24-hours (*r* (14) = 0.70, *p* < 0.01). IL-1β after LPS was not correlated with aromatase (*r* (14) = 0.35, *p* = 0.21).

### Aromatase assay & Immunocytochemistry

LPS injection increased aromatase activity in both males and females, with a main effect of treatment (*F* (1,24) = 7.48, *p* < 0.01), but with no main effect of sex or an interaction between the two (sex: *F* (1,24) = 2.90, *p* = 0.10; treatment x sex: (*F* (1,24) = 0.90, *p* = 0.35). See Fig. 4A.

To determine if LPS increased cell counts and the distribution of aromatase immunoproduct, an ICC on whole brain sections was performed on female tissue. Aromatase was detectable in several telencephalic and diencephalic nuclei across the brain^25^. More specifically, and as described previously, aromatase expression was observed at multiple telencephalic and diencephalic loci including the medial magnocellular nucleus of the nidopallium (mMAN), HPOA, hippocampus, caudomedial nidopallium, and nucleus taeniae, among others. Surprisingly, non-neuronal aromatase was not observed in any bird regardless of treatment (Fig. 5). The number of immunoreactive neurons/section in the hypothalamus was increased by LPS treatment relative to controls (*F* (1,4) = 2.87, *p* = 0.04). See Fig. 4.

## Discussion

In adult zebra finches of both sexes, we found that a peripheral injection of LPS: increased; (1) sickness-related behavior transiently, (2) cytokine expression rapidly and transiently and, (3) aromatase mRNA, activity and expression. Importantly, increases in aromatase were observed at a time when cytokine-expression had resolved to baseline levels. These data suggest that a peripheral inflammatory response is sufficient to induce neuronal aromatase in the brain.

To confirm that peripheral LPS injection had neural consequences, we measured indices of sickness behaviors and central cytokine mRNA. We found that both males and females ceased all locomotion 2-hours following LPS treatment. TNF-α and IL-1β mRNA are also elevated at this time point. However, at 24-hours post treatment, LPS birds exhibited normal levels of locomotor activity, and cytokine levels returned to baseline levels. Interestingly, aromatase mRNA and activity are elevated at this time point. This time course may suggest that peripheral inflammatory signaling may regulate neural aromatase expression via increases in central inflammation.

Inflammation increases aromatase expression in peripheral cells, including breast tissue^39–41^ and the endometrium^42^. A proposed mechanism is that PGE2 activates second messenger systems (cAMP and PKA) which increase CYP19 transcription, resulting in aromatase activity and E_2_ synthesis^39^. In postnatal rodents, inhibition or provision of PGE2 decreases or increases aromatase respectively *in vivo* ^30, 43^, but limited work has been done in the adult brain. The current study suggests that activation of inflammatory signaling via LPS increases aromatase expression at discrete neural loci. Furthermore, elevated TNF-α following LPS, but not IL-1β, is correlated with increased aromatase mRNA. This was not true of control animals. It is possible that TNF-α may be involved in the induction of aromatase. Ongoing work in our lab includes examining the signal(s) that are necessary for the induction of aromatase following insult.

A systemic inflammatory response produces cytokines, which circulate throughout the vasculature and communicate with the brain via the activation of central macrophages^33^. This can then cause a host of inflammatory signals, including proinflammatory cytokines and prostaglandins. The current study hypothesizes that this activation of central inflammatory signaling is sufficient to induce aromatase following a systemic immune response. This aromatization of local E_2_ content may serve to decrease inflammation in the brain. The consequent increase in neural E_2_ may be available to modulate a diverse set of physiological endpoints including but not limited to mitigation of chronic neuroinflammation, a particularly dangerous consequence of peripheral activation of the immune system and/or microbial invasion^44, 45^.

Previous work in our laboratory has found that central E_2_ provision via neural aromatization is neuroprotective in that it decreases apoptosis^46, 47^, gliosis^26^, inflammatory signaling^19^, and increases neurogenesis in the injured songbird brain^24^. This work, however, has focused on the induction of aromatase in non-neuronal, reactive astrocytes. In the present study, we were unable to detect any glial aromatase expression in birds treated with LPS or vehicle, strongly suggesting that the influence on aromatase transcription and activity and expression is a reflection of changes in neuronal aromatase. Indeed, LPS-treated birds had more immunoreactive neuronal profiles in the hypothalamus relative to vehicle-treated birds suggesting that activation of the peripheral immune system increases aromatization in central neurons. We chose to perform ICC on females since this sex has more rapid increases in aromatase expression in response to injury^48, 49^ and LPS treatment (current study). Although not statistically different from control females, LPS females had higher aromatase mRNA at two-hours compared to LPS males (p < 0.01). Current work in our lab is focusing on aromatase induction and expression in males. Similarly, we decided to measure aromatase activity at 24-hours, and not 2-hours, post-treatment. The 24-hour time point was chosen because both sexes had increases in aromatase mRNA compared to controls. However, although we detected no significant change in aromatase transcription two hours after LPS, allosteric changes in aromatase activity are possible. Future experiments will explore this possibility.

Although a limitation, we chose only analyze the hypothalamus for immunoreactive aromatase. Given that there was no detectable glial aromatase present in the telencephalon or diencephalon, we chose to conduct immunoreactive cell counts in the mPOA, an area with high constitutive amounts of neuronal aromatase^25^. We hypothesize that increased immunoreactive neuronal aromatase would also be present in the telencephalon, which would correspond to mRNA and activity data. The current study failed to detect any glial aromatase, however, it is possible that varying intensity of staining, time point, or the dosage might enable the visualization of glial aromatase. Current experiments are exploring this possibility.

An increase in aromatase mPOA neurons is perhaps surprising, giving the historical association of the POA with socio-sexual behaviors, and immune challenge is linked to the decrease of behaviors not related to immediate survival^50^. Although aromatase and E_2_ in the POA are known for the maintenance of socio-sexual behavior, current data suggests that higher concentrations of E_2_ may inhibit socio-sexual behavior. It is also possible that the increases in aromatase, and presumably E_2_, serve to decrease neurotrauma. As with glial aromatase, increases in neuronal aromatase may be neuroprotective in the inflamed brain. E_2_ inhibition and replacement intensify and diminish microglia activation^21, 51^, and NF-κB cytoplasmic transport^52^ LPS treatment in cell culture, respectively. This suggests that perhaps the induced aromatase and E_2_ following LPS may serve to decrease chronic neuroinflammatory signaling.

Neural increases in cytokine expression and resting behavior following LPS treatment suggest an increase in central inflammation. LPS has routinely and reliably been shown to stimulate inflammatory signaling, via TLR-4 activation and thereby induces the release of several proinflammatory cytokines, microglia activation, and neurotoxic factor release^53–55^. In zebra finches, LPS increases free radicals^56^ and plasma cytokine levels^57^ suggesting a conservation of inflammatory pathways across vertebrate species. It is possible that the induced inflammation results in aromatase expression in the zebra finch. Inflammation has been shown to regulate peripheral and central aromatase expression^30, 39–43^. A peripheral LPS injection activates central inflammatory signaling, such as increases in cytokines and prostaglandins^58^. Given previous literature, we predict that the cytokine induction of PGE2 may result in aromatase induction and consequent E_2_ synthesis following LPS treatment. Current work in our lab is exploring this hypothesis.

Our lab has previously detected glial aromatase following a penetrating brain injury^19, 22, 26, 47, 59^ and following inflammagen application directly to the brain^31^. However, a peripheral treatment of an inflammagen was not able to induce glial aromatase (present study). It is possible that an additional signal associated with central injury may be needed for the induction of glial aromatase. This hypothesis is currently being explored in our laboratory, as are other time points after LPS treatment.

In conclusion, the current study suggests that peripheral activation of inflammatory signaling is sufficient to induce aromatase activity in the brain. Therefore, inflammation may regulate aromatase and therefore estradiol induction. Current work is exploring the consequences of this induction and assessing if it is neuroprotective.

## Methods

Subjects consisted of male (n = 24) and female (n = 30) adult zebra finches (*Taenioygia guttata*) housed at American University in same-sex flight cages. Animals were maintained on a 12:12 hour light-dark cycle in a temperature and humidity controlled room (72° ± 2). Food and water were provided *ad libitum*. The day before the experiment, subjects were chosen at random and placed in individually housed condos. Subjects remained in auditory contact of conspecifics but were not in visual contact. All experiments were carried out in accordance with relevant guidelines and regulations, and American University Institutional Animal Care and Use Committee (IACUC) approved all experimental protocols.

### Treatment and experimental design

**Figure 1:**
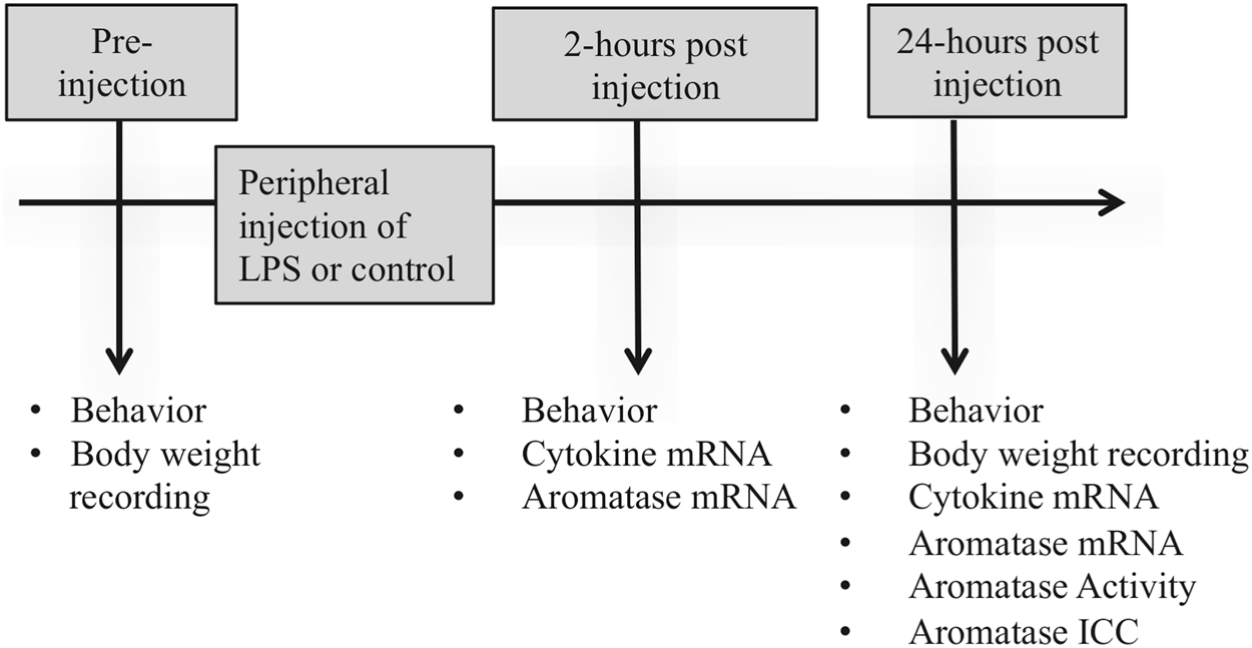
Schematic depicting the experimental design of the current study including treatment, behavioral observations, cytokine expression, and the expression of aromatase.

Birds were given an injection of 100 μL of LPS (0.3 mg mL^−1^ ^57, 60^) or vehicle (1X PBS, 0.01 M, pH = 7.4). Locomotor behaviors were recorded immediately prior, two hours post- and 24 hours post-injection. Body weight was also measured prior to injection and 24 hours post-injection. Subjects were either sacrificed 2- (n = 24) or 24-hours (n = 24) post injection. The neural transcription of cytokines and aromatase was measured at both time points. Twenty-four hours post-injection we also measured the activity of the aromatase enzyme in telencephalic homogenates and measured the expression of aromatase using immunocytochemistry (ICC). See Fig. 1 for experimental design.

**Figure 2:**
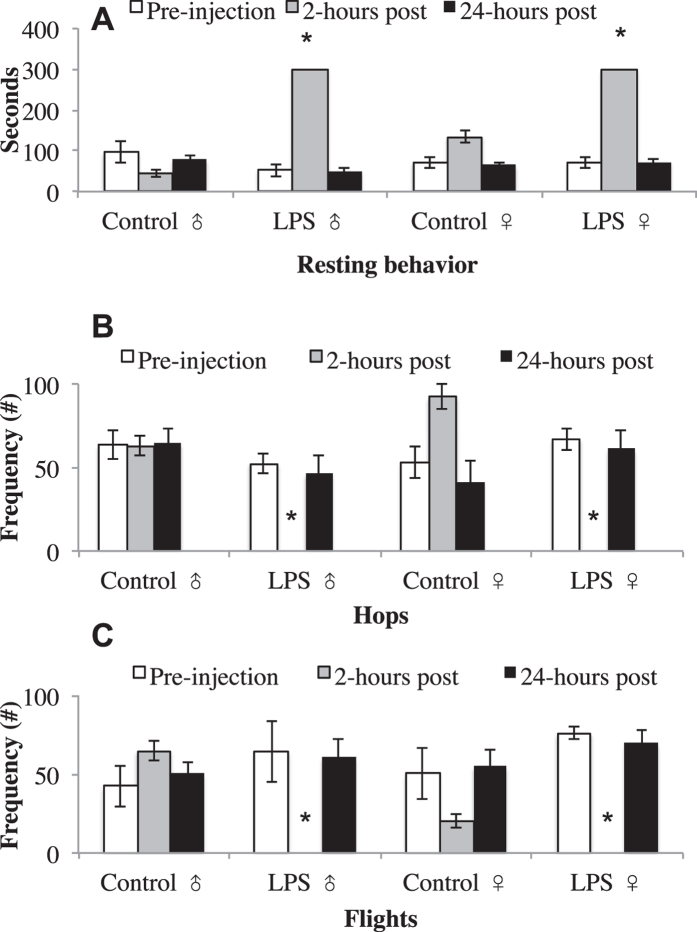
Locomotor behavior as a function of treatment. Mean (+SEM) of time subjects spent resting (**A**), number of hops (**B**), and number of flights (**C**) pre-, 2-hours post-, and 24-hours post-treatment. LPS birds at 2-hours post treatment spent more time resting, and had less locomotor behavior, than control subjects and pre-treatment observations. There were no differences in resting behavior 24-hours post injection. *p < 0.05.

**Figure 3:**
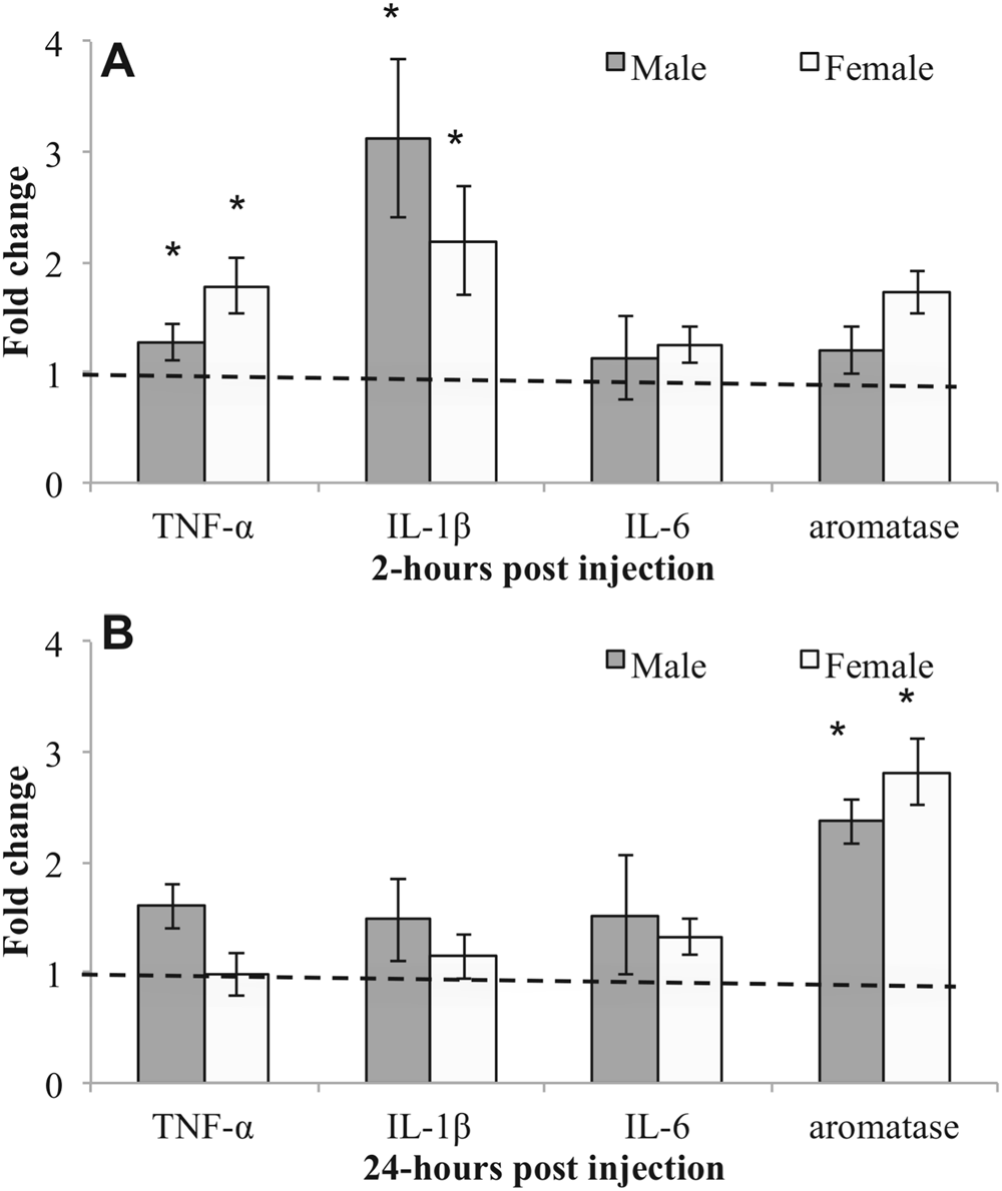
Levels of cytokine and aromatase mRNA relative to glyceraldehyde 3-phosphate dehydrogenase (GAPDH; fold change values) in adult male and female zebra finches. Following LPS-treatment, IL-1β mRNA is upregulated in both sexes and TNF-α in higher in females treated with LPS relative to controls. However, levels of both transcripts are back at control levels 24-hours following LPS, a time when aromatase mRNA is elevated in LPS birds relative to controls. The dashed line represents the level of each transcript normalized to “1,” in vehicle-treated controls, regardless of the levels across individual cytokines and aromatase. *p < 0.05.

**Figure 4:**
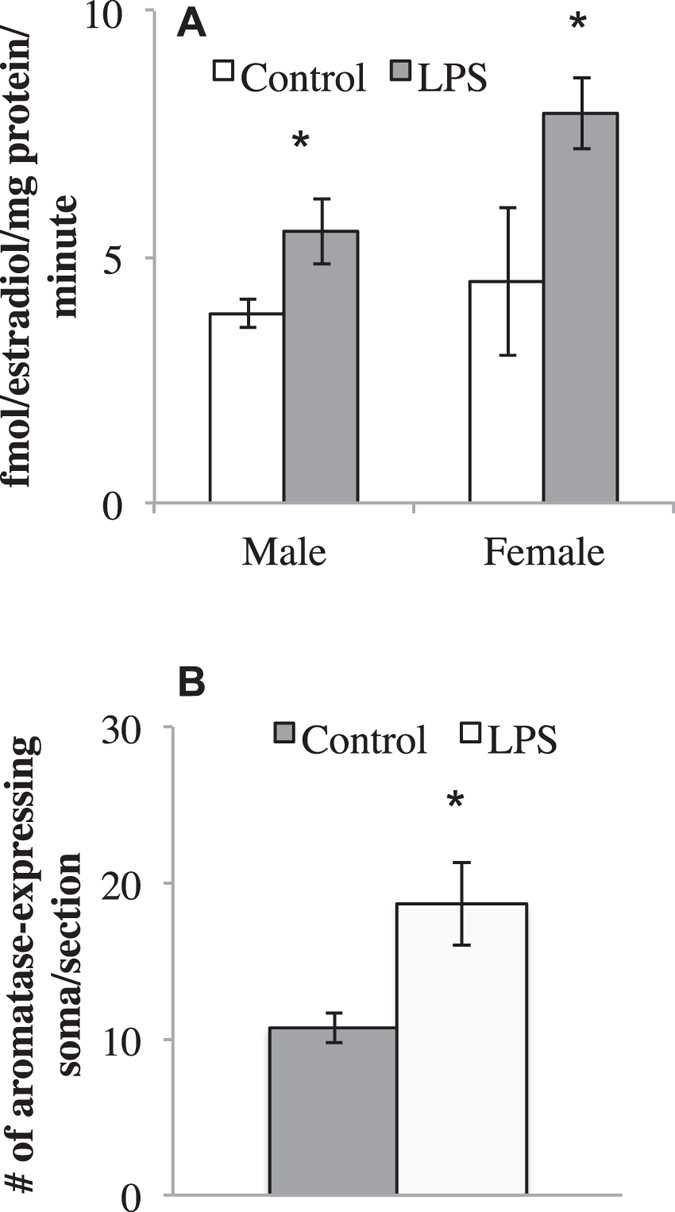
Levels of aromatase activity (**A**) and number of aromatase positive cells across the HPOA (**B**). 24-hours post-LPS treatment, aromatase activity is elevated compared to control subjects. The number of immunoreactive neurons/section in the hypothalamus was increased by LPS treatment relative to controls. *p < 0.05.

**Figure 5:**
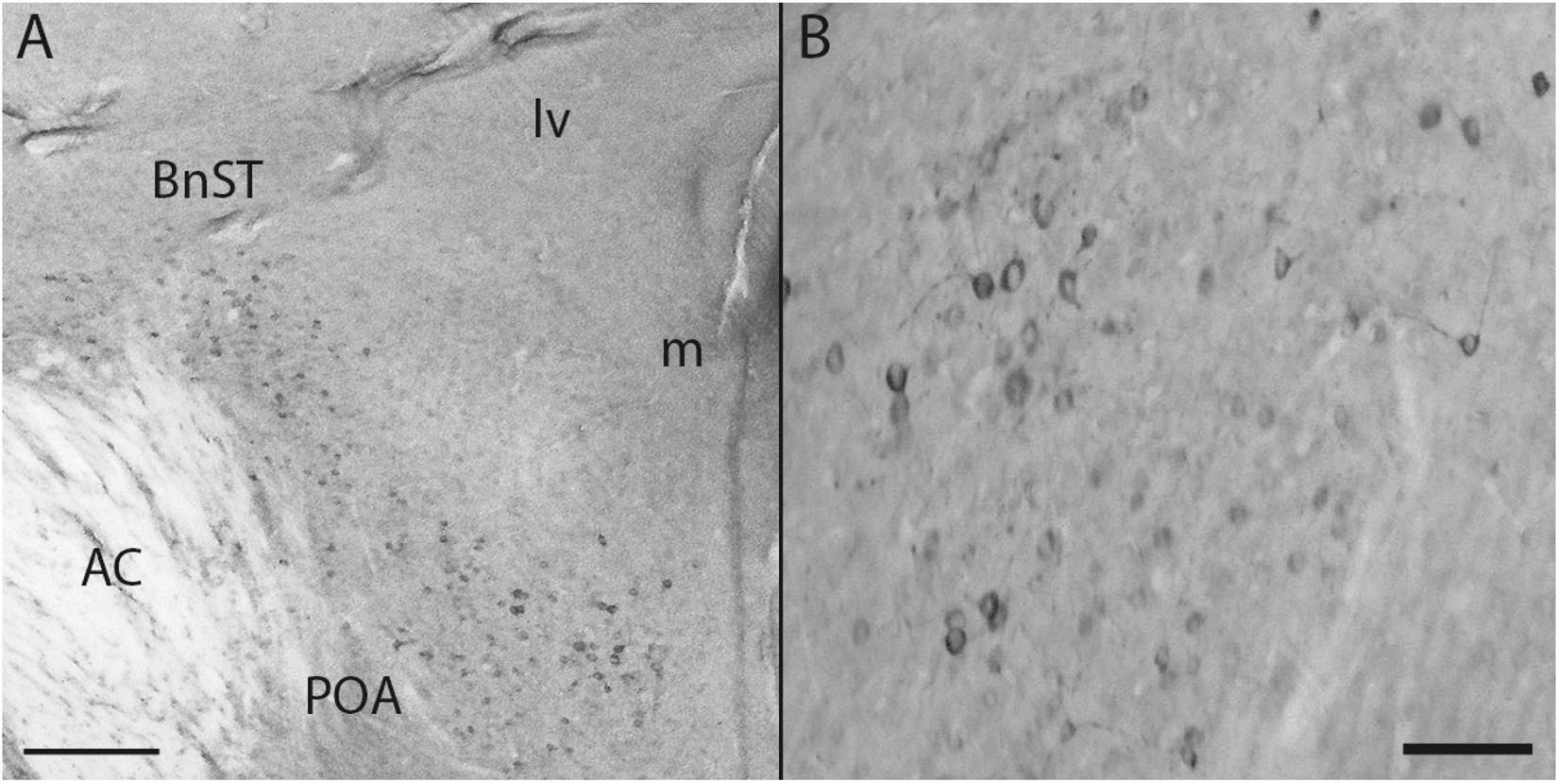
Low-power (**A**) and high-power (**B**) photomicrographs depicting aromatase-positive neurons following LPS treatment. Note the presence of neuronal, but not glial aromatase expression in the diencephalon and telencephalon. Bed nucleus of the stria terminalis (BnST), preoptic area (POA), anterior commissure (AC), lateral ventricle (lv) and midline (m). Magnification bars = 200 μm (**A**) and 75 μm (**B**).

### Body Weight

To measure the effectiveness of peripheral immune challenge, we measured body weight prior to and post-treatment. On day 1 of the experiment, subjects were removed from individually housed cages and weighed prior to the first injection. Subjects were re-weighed prior to euthanasia at 24-hours post injection and the change in body weight computed for statistical analysis.

### Behavior

Behavior of subjects was recorded in one pre- and two post-treatment observations. On the day of the experiment at 08:00 hours, each subject (n = 24/sex) was individually recorded via camera for ten minutes, and the number of hops and flights were counted during the first five-minute period^57^. The latter five minutes were recorded to observe the duration the subject spent resting. All behavior was recorded via camera by an investigator who administered treatment injection. Recorded behavior was given to a trained, blinded observer to analyze.

### Tissue Collection

Two- (n = 24) or 24-hours (n = 30) post injection, birds were decapitated, and the telencephalon was dissected. The left and right hemispheres were separated from each other, and the cerebellum was discarded. Telencephalic tissue was weighed and then stored at −80 **°**C until further processing. One telencephalic hemisphere was used for qPCR, and the opposite hemisphere was used for the aromatase assay. We alternated between right or left telencephalic lobes to counterbalance for potential hemispheric differences.

### Quantitative Polymerase-Chain Reaction

One telencephalic hemisphere (n = 48) was homogenized in 500 uL 0.1 M PB and total RNA extracted. RNA was extracted using RNeasy Mini Extraction Kit (Qiagen) according to manufacturer’s instructions, and the quality of RNA extraction was analyzed on a ND-100 spectrophotometer. Only extracts that exceeded a 260/280 ratio of 1.8 were used.

Primers for TNF-α, IL-1β, IL-6, aromatase, and the housekeeping gene GAPDH, were designed against the zebra finch genome^19^. Recent work has suggested GAPDH may not be the best housekeeping gene for this species^61^. However, in the current study, GAPDH did not significantly vary across treatment groups (average + SEM of ΔCt for controls 15.69 ± 0.25 vs. LPS 15.55 ± 0.54; F (1, 38) = 1.18, *p* = 0.29). Therefore, we felt confident in normalizing our gene targets against this housekeeping gene. Primers were validated in our lab, and validation included: PCR to confirm band size, sequencing (Beckman Coulter Genomics), and template and primer serial dilutions, and efficiency confirmation of 100%^19^.

The expression of target transcripts was analyzed on a 96-well plate and Syber Green One-Step qPR-PCR kit (Invitrogen). Each sample was run in triplicate, and each well had 75 ng of total RNA in a 15-μL reaction. A no template control and no transcriptase control were run on each plate to assess the possibility of containments. An inter-run calibrator was run on each plate to allow for the comparison across runs.

### Aromatase assay

Aromatase activity was measured by quantifying the amount of titrated water produced by the aromatization of [1β-3H] androstenedione according to Roselli & Resko^62^, with little modification. The influence of LPS on aromatase activity was measured 24-hours post treatment, because detectable changes in the aromatase transcript were only apparent at this time-point (see Fig. 3). Briefly, one telencephalic hemisphere (n = 24) was homogenized in 500 μL of TEK buffer (150 mM KCl, 10 mM Tris, and 1 mM Na-EDTA, pH = 7.2). All assay tubes were kept on ice unless otherwise specified. Equal aliquots (50 uL) of TEK buffer, diluted homogenate, androstenedione (specific activity = 24.0 Ci/mMol) and NADPH were added to a reaction tube and incubated at 37 °C for 15 minutes. Samples were run in duplicate, and a third tube served as a control to which 50 μM of fadrozole diluted in TEK buffer was added in place of TEK buffer. Fadrozole is a well-characterized and potent aromatase inhibitor; in this instance, fadrozole was used as a within sample control as it blocks all available aromatase in each sample of tissue and the radioactivity left in the scintillation vial following washouts serves as a “blank” for each of the given samples. The reaction was stopped by adding 10% trichloroacetic acid. These assay tubes were then centrifuged at 4 °C for 20 minutes at 5,000 RCF. Supernatant was removed from the tubes and added to flow-through columns made by Pasteur pipettes plugged with one 3 cm glass bead, and filled with 1.4 mL dowex cation exchange resin (AG 50 W-X4, 100–200 mesh Bio-Rad). The columns were then rinsed with dH2O and all effluent was collected in 20 mL scintillation vials. Finally, 10 mL Ecoscint A (National Diagnostics) was added and radioactivity emitted from the vials was counted for 5 minutes after a minimum 4-hour incubation on a Beckman LS-6500 scintillation counter. A Bradford protein assay (ThermoFisher Scientific) was used to determine the exact amount of protein in each reaction tube. Aromatase activity was expressed as femtomoles of estradiol per mg protein per minute, after blank subtraction and accounting for the specific activity, the recovery yield of the column, the fraction of tritium with the β-decay.

### Immunocytochemistry for aromatase

In order to determine the neural distribution and cellular identity, and to obtain a semi-quantitative measure of aromatase expression following LPS injection, we used immunocytochemistry with a specific antibody using previously published protocols^22, 25, 31, 47, 63^. The influence of LPS on aromatase expression was measured 24-hour post treatment because aromatase transcript was only increased at this time-point (see qPCR results below). Whole brain sections from an additional six adult females were removed 24-hours post treatment injection. Females were used for this part of the study since this sex is known to have a more robust response of aromatase to brain perturbation and to the current data suggested a marginally quicker response to LPS (present study; see Results)^49^.

Tissue was fixed in 5% acrolein (diluted in 0.1 M PB and added to distilled water; Electron Microscopy Sciences) and cut on a vibratome after being gel embedded (8% gel from 300 bloom gelatin; VWR). Standard protocols for free floating sections were used to stain for aromatase using a specific aromatase antibody created against the c-terminal of zebra finch aromatase (AZAC)^22, 25, 26, 31, 47^. Antibody validation included: variation of antibody concentration for optimal staining, runs with a no primary control, amplicon confirmation via Western Blot analysis, and presence of staining in ovarian tissue, and absence of staining in pectoral muscle^25^.

In this study, each ICC run included sections from one experimental and one control bird (total of three ICC runs). Briefly, sections were washed several times in 0.1 M PB, rinsed in 0.036% H_2_0_2_ (ten minutes), washed, and incubated in normal goat serum (60 minutes). Then, sections were placed in primary with the antibody AZAC (1:2500) for 48 hours at 4 °C. Sections were then washed, incubated in goat anti-rabbit (60 minutes). Following secondary incubation, sections were washed and incubated in 1:200 avidin-biotin complex (Vectastain) for 60 minutes. Following a wash, aromatase immunoproduct was visualized using a chromagen-peroxide solution. Sections were then mounted on slides and then coverslipped after dehydrated through graded alcohols (70, 95, 95, 100, 100%). Slides were examined on a Nikon eclipse E100M to determine the presence and distribution of aromatase protein. Three representative micrographs from the preoptic area (POA) were examined at 400X and images were captured for quantification. More specifically, images were collected from the medial portion of the POA, where a bilateral nucleus of aromatase-expressing neurons is located^25^. This nucleus appears in the anterior hypothalamus ventral to the anterior commissure and dorsal to the third ventricle^25^. To avoid re-sampling or capturing overlapping portions of the nucleus, only a single image centered on one side of the brain nucleus was captured, and successive images were separated by at least 300μm by sampling in the center of the brain nucleus on the contralateral side of the midline or in successive coronal sections. An experimenter blind to treatment conditions counted the total number of labeled cells in each brain section (anterior brain, mid-brain, and posterior). Counts were restricted to only neuronal profiles with clear, unstained nuclei.

### Statistics

#### Body weight

To analyze the effect of LPS on body mass, we weighed subjects immediately before and 24-hours post treatment. Mass loss was obtained by subtracting mass at day 2 minus mass at day 1. We conducted statistical analyses on these values. A two-way analysis of variance used with treatment as sex as main variables.

#### Behavior

Each behavior (hops, flights, and resting behavior) was analyzed individually by time point (pre-injection, post-injection, and 24-hours post-injection). Three two-way analysis of variance with treatment and sex as the main variables were performed. A Tukey-Kramer test was used to analyze the source of significant main effects, and Fisher LSD pairwise comparisons were used to assess the source of significant interactions.

#### Quantitative Polymerase-Chain Reaction data

The delta threshold cycle number (ΔCt) method was used for quantification using the detection threshold (Ct) for each target gene less the Ct for the housekeeping gene, glyceraldehyde 3-phosphate dehydrogenase (GAPDH). All statistical analyses are only performed on ΔCt. For all graphs, fold change data are presented (2^−Δ(ΔCT)^). This normalizes control condition ΔCt value to 1^19, 31, 32, 64^. In this case, male controls served to normalize male LPS data, and female controls normalized female LPS data. This fold change calculation is only for the presentation of graphical data. To assess baseline differences across control groups, please refer to ΔCt values in Table 1.

ΔCt values for TNF-α, IL-1β, IL-6, and aromatase were analyzed separately using a two-way analysis of variance with treatment and sex as the main variables. Time points (2 and 24-hours post treatment) were analyzed separately to avoid excessive and meaningless comparisons. Sex, instead of time, was chosen as a main variable due to previous studies showing a sex differences the pattern of cytokines following injury^19^ and a sex difference in the induction of aromatase^49^. A Tukey-Kramer test was used to analyze the source of significant main effects and Fisher LSD pairwise comparisons were used to assess the source of significant interactions. We also performed Pearson correlations on ΔCt values to explore the relationship cytokine data and aromatase, to test if there is a positive correlation of cytokine mRNA following 2-hours post-treatment and aromatase mRNA 24-hours post treatment.

#### Aromatase Assay

A log transformation was done on aromatase assay data due to an unequal variance across groups. We then performed a two-way analysis of variance with sex and treatment as the main variables. Although statistics were conducted on log-transformed data, the untransformed data are presented in Fig. 4. A Tukey-Kramer test was used to analyze the source of significant main effects and Fisher LSD pairwise comparisons were used to assess the source of significant interactions.

#### Aromatase Immunocytochemistry

A one-way analysis of variance was conducted with treatment as the main variable. For each animal, we obtained an average of aromatase expression across anterior, middle, and posterior sections of the hypothalamus. This gives us a representation of aromatase expression in the hypothalamus of control and LPS-treated birds.
